# Mechanistic insights into mRNA 3′-end processing

**DOI:** 10.1016/j.sbi.2019.08.001

**Published:** 2019-12

**Authors:** Ananthanarayanan Kumar, Marcello Clerici, Lena M Muckenfuss, Lori A Passmore, Martin Jinek

**Affiliations:** 1 MRC Laboratory of Molecular Biology, Cambridge CB2 0QH, United Kingdom; 2 Department of Biochemistry, University of Zurich, Winterthurerstrasse 190, CH-8057 Zurich, Switzerland

## Abstract

The polyadenosine (poly(A)) tail found on the 3′-end of almost all eukaryotic mRNAs is important for mRNA stability and regulation of translation. mRNA 3′-end processing occurs co-transcriptionally and involves more than 20 proteins to specifically recognize the polyadenylation site, cleave the pre-mRNA, add a poly(A) tail, and trigger transcription termination. The polyadenylation site (PAS) defines the end of the 3′-untranslated region (3′-UTR) and, therefore, selection of the cleavage site is a critical event in regulating gene expression. Integrated structural biology approaches including biochemical reconstitution of multi-subunit complexes, cross-linking mass spectrometry, and structural analyses by X- ray crystallography and single-particle electron cryo-microscopy (cryoEM) have enabled recent progress in understanding the molecular mechanisms of the mRNA 3′-end processing machinery. Here, we describe new molecular insights into pre-mRNA recognition, cleavage and polyadenylation.

## Highlights

• Integrated structural biology approaches have provided new insights into the mechanism of eukaryotic mRNA 3′-end processing.
• The polymerase modules of yeast and human cleavage and polyadenylation factors share a conserved architecture.
• CryoEM structures of human CPSF have revealed the mechanism of AAUAAA polyadenylation signal recognition.
• Cleavage and polyadenylation of mRNA 3′-ends likely involves a dynamic assembly of CPF/CPSF and accessory factors.

**Current Opinion in Structural Biology** 2019, **59**:143–150

This review comes from a themed issue on **Protein nucleic acid interactions**

Edited by **Frédéric H-T Allain** and **Martin Jinek**

For a complete overview see the Issue and the Editorial

Available online 6th September 2019

**https://doi.org/10.1016/j.sbi.2019.08.001**

0959-440X/© 2019 MRC Laboratory of Molecular Biology. Published by Elsevier Ltd. This is an open access article under the CC BY license (http://creativecommons.org/licenses/by/4.0/).

## Introduction

**Figure 1:**
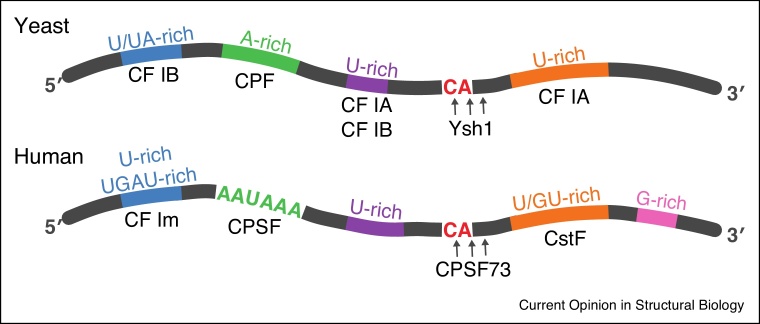
Polyadenylation signals and RNA recognition sites of the 3′-end processing machinery in yeast and human pre-mRNAs. Protein complexes that are proposed to recognize specific cis-acting elements within the polyadenylation signal are listed below their consensus sequences. Arrows indicate cleavage positions.

Most eukaryotic pre-mRNAs are capped at their 5′-end, spliced at intronic sites, and polyadenylated at their 3′-end before they are exported from the nucleus as mature mRNAs. Each of these modifications is carried out by a set of conserved and highly regulated multi-protein complexes. The 3′-end processing machinery co-transcriptionally monitors nascent transcripts for specific sequences (Figure 1) and, upon recognition of the polyadenylation site (PAS), cleaves the pre-mRNA and adds a poly(A) tail to the newly generated 3′-end [1]. The 3′-end processing machinery also triggers transcription termination. To co-ordinate these functions, it contains three different enzymatic activities — endonuclease, poly(A) polymerase, and protein phosphatase. Since the cleavage event defines the 3′-end of the transcript, and consequently the 3′-untranslated region (3′-UTR) of the future mRNA, understanding how RNA is specifically recognized is of key importance.

The large number of protein factors involved and the dynamic nature of their interactions pose challenges in understanding the molecular mechanisms of mRNA 3′-end biogenesis. Until very recently, high-resolution structures were available for only a few of the proteins involved [2,3]. Ysh1/CPSF73 had been identified as the 3′-endonuclease and its crystal structure was determined [4^•^]. Crystal structures were also available for the poly(A) polymerase Pap1/PAP [5,6]. Together, these structures provided insights into the enzymatic mechanisms but they did not explain how pre-mRNAs are specifically recognized and how the different enzymatic activities are coordinated.

Recent developments in structure determination using electron cryo-microscopy (cryoEM), native mass spectrometry and cross-linking mass spectrometry (XL-MS), combined with next-generation sequencing-based functional methods, have facilitated progress in understanding the molecular mechanisms of pre-mRNA processing. In this review, we outline molecular details of the core machinery that mediates pre-mRNA cleavage and polyadenylation, focusing on insights obtained within the last two years. Structures and mechanisms of other components of the mRNA 3′-end processing machinery have been covered extensively by other reviews [2,3].

## Composition and functions of 3′-end processing machinery

Specific and efficient mRNA 3′-end processing is coordinated by the concerted action of a large number of conserved proteins (Table 1). Many components were first identified by cell fractionation studies, both from yeast and human extracts [1], and proteomic studies further defined the components of affinity-purified native complexes from *Saccharomyces cerevisiae* [7, 8, 9, 10]. The yeast machinery comprises three factors: cleavage and polyadenylation factor (CPF), which contains the enzymatic activities, cleavage factor (CF) IA and CF IB [1,11]. More recently, affinity-purification of an endogenous, intact CPF complex from yeast enabled thorough analysis of its composition, stoichiometry, and subunit assembly [12^••^]. Specifically, native mass spectrometry defined the protein–protein interaction network within the complex, revealing that the CPF subunits are assembled into three modules, each based on one of the enzymatic activities: endonuclease, polymerase or phosphatase (Table 1). The interaction map of yeast CPF subunits enabled-specific cleavage and polyadenylation to be reconstituted from complexes of purified recombinant yeast proteins [12^••^,13^••^]. *In vivo* transcriptome-wide mapping of yeast pre-mRNA biogenesis factors previously showed that CPF binds AU-rich elements near the cleavage site [14]. Still, isolated CPF is not substantially active without the accessory factors CF IA and CF IB, which contribute to RNA recognition and activation of the nuclease [3,15]. Both CF IA and CF IB bind-specific RNA sequences near the poly(A) site (Figure 1) [3,15].

**Table 1:** Yeast and human mRNA 3′-end processing machinery

Yeast complex	Module	Yeast protein^a^	M.W. (kDa)	Human Protein^a^	M.W. (kDa)	Human complex	% Sequence identity (similarity)	Proposed role
CPF	Polymerase^b^	**Pap1**	65	**PAP**	83		40 (59)	**Poly(A) polymerase**
		Cft1/Yhh1	153	CPSF160/CPSF1	161	CPSF	17 (34)	Scaffold
		Pfs2	53	WDR33	146		37 (57)	Scaffold, RNA binding
		Fip1	36	hFip1	67		22 (34)	Binds Pap1
		Yth1	25	CPSF30/CPSF4	30		36 (51)	RNA binding
	Nuclease^b^	Cft2/Ydh1	96	CPSF100/CPSF2	88		22 (41)	(Pseudo-)endonuclease
		**Ysh1/Brr5**	88	**CPSF73/CPSF3**	77		44 (65)	**Endonuclease**
		Mpe1	50	RBBP6	202		28 (48)	RNA binding
	Phosphatase	Pta1	89	Symplekin	141		17 (37)	Scaffold
		Ref2	60	n.d.	–		–	Regulates Glc7
		Pti1	47	n.d.	–		–	Scaffold
		Swd2/Cps35	37	WDR82	35		34 (52)	Transcription termination
		**Glc7**	36	**PP1A**	38		85 (93)	**Phosphatase** of Pol II CTD
		**Ssu72**	24	**SSU72**	23		44 (65)	**Phosphatase** of Pol II CTD
CF IA		Rna14	78	CstF77/CSTF3	83	CstF	26 (42)	Stoichiometry of 2, scaffold
		Rna15	33	CstF64/CSTF2	61		27 (41)	Stoichiometry of 2, RNA binding
		n.d.		CstF50/CSTF1	48		–	
		Clp1	50	hClp1	48	CF IIm	27 (44)	RNA kinase in human
		Pcf11	72	hPcf11	173		21 (32)	Binds Pol II
CF IB		Hrp1	60	n.d.	–		–	RNA binding
		n.d.		CFIm25/CPSF5/NUDT21	26	CF Im	–	RNA binding
		n.d.		CFIm68/CPSF6	59		–	RNA binding
		n.d.		CFIm59/CPSF7	52		–	RNA binding

n.d., none detected.

a Enzymes in bold.

b Yeast polymerase and nuclease modules comprise the ‘Core CPF’ (CPF_core_).

The human mRNA 3′-end processing machinery comprises the cleavage and polyadenylation specificity factor (CPSF), cleavage stimulation factor (CstF), cleavage factors Im (CF Im) and IIm (CF IIm), and poly(A) polymerase (PAP) [3]. These factors include many orthologs of the yeast machinery (Table 1), but a fully active human complex has not yet been reconstituted from recombinant proteins. Whether the mammalian CPSF assembles in a similar modular fashion as CPF has not yet been confirmed but it appears to be functionally equivalent to the yeast core CPF complex (CPF_core_) comprising the nuclease and polymerase modules [13^••^,16^•^]. Some of the mammalian components, including WDR33 and Fip1, are much longer than the yeast subunits. Unlike in yeast, human PAP is not a stable component of the complex. A proteomic study of the human pre-mRNA 3′-end processing complex identified ∼85 associated proteins [17^•^], but the functions of many of these have not been thoroughly investigated.

CPSF specifically recognizes a hexanucleotide AAUAAA motif within the polyadenylation signal (PAS), directing cleavage of the pre-mRNA 10–30 nucleotides downstream [16^•^,18^•^] (Figure 1). Transcriptome-wide studies of mammalian mRNA polyadenylation have shown that the AAUAAA PAS motif is highly conserved [19, 20, 21]. Recognition of upstream UGUA-containing sequences (USEs) and downstream G-rich and GU-rich sequences (DSEs) by the CF Im and CstF complexes, respectively, contributes to selection of the cleavage site and ensures efficient pre-mRNA recognition and cleavage [3].

## Molecular architecture of the polymerase module

**Figure 2:**
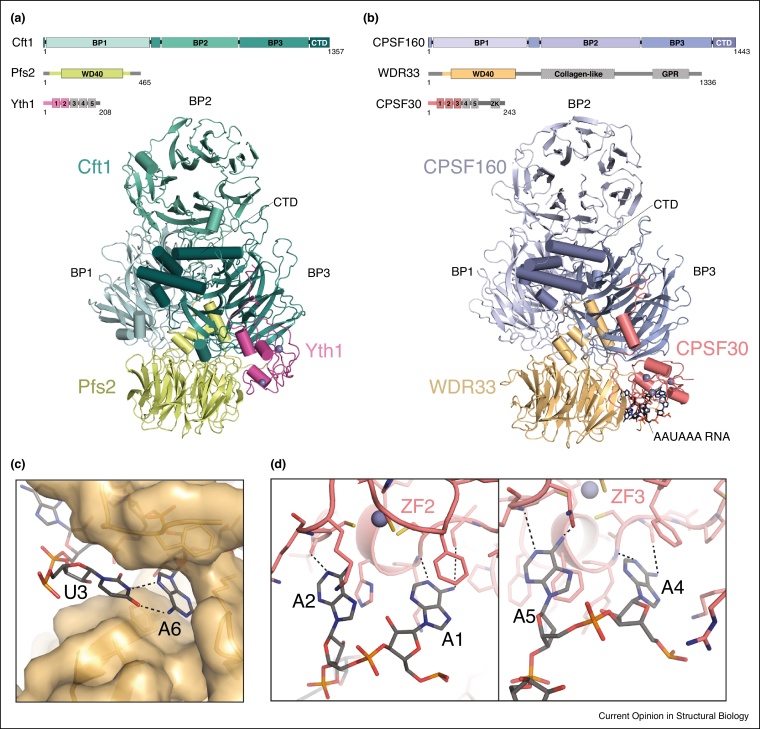
Molecular architecture of the structural scaffolds of yeast CPF/human CPSF and mechanism of polyadenylation signal recognition. **(a)** Structure of the yeast Cft1–Pfs2–Yth1 complex (PDB code 6EOJ). **(b)** Structure of the human CPSF160–WDR33–CPSF30 complex bound to AAUAAA PAS RNA (PDB code 6FUW). Protein regions not observed or present in the shown structures are indicated in grey in the domain architecture diagrams above each structure. **(c)** Specific recognition of the Hoogsteen base pair formed by U3 and A6 of the AAUAAA PAS by a hydrophobic cavity formed by the N-terminal extension of WDR33. **(d)** Molecular details of AA dinucleotide recognition by CPSF30 zinc finger (ZF) domains 2 (left panel) and 3 (right panel). Hydrogen-bonding interactions are depicted with dashed lines.

Guided by the protein–protein interaction network map, a ∼200-kDa recombinant four-subunit Cft1–Pfs2–Yth1–Fip1 complex of the polymerase module was recently analyzed using cryoEM. This resulted in a 3-D reconstruction comprising Cft1, Pfs2, and zinc finger (ZF) domains 1 and 2 of Yth1 [12^••^]. Cft1 contains three seven-bladed beta-propeller domains (BP1, BP2, BP3) followed by a C-terminal helical domain, and is intimately associated with Pfs2, a WD40-protein composed of a beta-propeller domain and an N-terminal protrusion that inserts into the cavity formed by Cft1 BP1 and BP3 (Figure 2a). The Cft1–Pfs2 interface is extensive, burying >4200 Å^2^ surface area, and is highly conserved. The same interaction mode was observed in X-ray crystallographic and cryoEM structures of the orthologous human CPSF160–WDR33 heterodimer [22^••^,23^••^,24^••^]. The triple beta-propeller domain architecture of Cft1/CPSF160 is structurally homologous to that of DNA Damage Binding protein 1 (DDB1) [25] and splicing factor SF3b subunit Rse1 [26] (SF3b130 in human) despite low sequence conservation (∼15%). Interestingly, the interaction of DDB1 with its binding partner DDB2 has some similarities to the Cft1–Pfs2 and CPSF160–WDR33 interactions but the details of the subunit contacts are not conserved.

The Cft1–Pfs2 heterodimer constitutes a rigid core platform of the polymerase module which binds an N-terminal extended region and ZF1–2 of Yth1 (Figure 2a). ZF3–5 of Yth1 and the entirety of Fip1 are not observed in the cryo-EM reconstruction, indicating that they are flexibly tethered to Cft1–Pfs2, at least in the absence of other CPF subunits and/or RNA. Fip1, an intrinsically disordered protein, forms a physical connection between the C-terminal region of Yth1 and Pap1 [12^••^,^27^]. In an analogous manner, the human CPSF160–WDR33 heterodimer forms the structural scaffold of CPSF (Figure 2b), while CPSF30 bridges the interaction between CPSF160–WDR33 and Fip1, based on XL-MS analysis and co-precipitation experiments [22^••^]. Consistent with its absence in yeast Yth1, the CPSF30 zinc knuckle (ZK) domain is not required for complex assembly and its function is hitherto unknown [22^••^].

## Recognition of the polyadenylation signal

Similar to the yeast polymerase module, human CPSF160, WDR33, CPSF30 and Fip1 form a stable heterotetrameric core complex that recognizes the AAUAAA polyadenylation signal motif and recruits PAP [16^•^]. The molecular mechanism of PAS RNA recognition by the mammalian 3′-end processing machinery was recently revealed by two independently determined cryoEM structures of the human CPSF160–WDR33–CPSF30–Fip1 complex bound to AAUAAA-containing RNA. The overall architecture of the complex is highly similar to the yeast Cft1–Pfs2–Yth1–Fip1 structure. However, in the presence of the PAS RNA, the CPSF30 ZF3 domain becomes ordered (Figure 2b). PAS recognition is mediated by the CPSF30 ZF2 and ZF3 domains and WDR33 (Figure 2c and d) [23^••^,24^••^], as indicated by prior studies [16^•^,18^•^,^27^]. Although CPSF160 was previously implicated in PAS recognition [28], the RNA is not contacted by CPSF160 directly.

Recognition of the AAUAAA motif by CPSF30 involves bending of the RNA sugar-phosphate backbone into an S-shaped conformation, stabilized by extensive interactions with WDR33, in particular with its N-terminal extension that encircles the RNA. The kinked RNA conformation is further stabilized by an intramolecular Hoogsteen base pair formed between nucleotides U3 and A6 (Figure 2c). The adenosine dinucleotides in positions 1–2 and 4–5 of the AAUAAA motif are bound by the CPSF30 ZF2 and ZF3 domains, respectively (Figure 2d). Each nucleotide base is inserted into a pocket of the respective ZF domain and stacks with a conserved aromatic residue. Nucleotides A1, A4 and A5 are almost invariant in the PAS and each adenine base is recognized by two base-specific hydrogen-bonding interactions with the N1 and N6-amino groups. In contrast, the adenine base of the more variable nucleotide A2 interacts with CPSF30 via a single hydrogen-bonding contact.

Contrary to the adenosine dinucleotides A1–A2 and A4–A5, the U3–A6 Hoogsteen base-pair is not recognized by base-specific interactions, but is sandwiched between two conserved phenylalanine residues which stabilize the base-pair with π–π stacking interactions. The shape of the hydrophobic pocket formed by WDR33 to accommodate the U3–A6 pair is not compatible with other purine-pyrimidine combinations (Figure 2c). Thus, the intricate and specific network of molecular interactions established between CPSF30, WDR33 and all six nucleotide positions of the PAS provides a rationale for the widespread conservation of the AAUAAA motif revealed by transcriptome-wide mapping of mammalian mRNA polyadenylation sites [19, 20, 21]. In agreement with this, single-base substitutions in the PAS AAUAAA motif can result in a substantial reduction in the RNA binding affinity of the CPSF complex [22^••^] and in deficient mRNA processing in human diseases such as α-thalassemia and β-thalassemia [29,30]. The structural insights suggest that non-canonical PAS motifs with one or more base substitutions [21] may function as weak polyadenylation sites: lower affinity of the non-canonical PAS motif for the CPSF complex may result in decreased use of that site. In the context of alternative polyadenylation, the weak affinity of non-canonical PAS motifs is likely compensated for by additional upstream and downstream *cis*-acting elements that enhance CPSF-binding under specific conditions or in response to specific signals.

In yeast, the polyadenylation signals (also termed positioning elements) are less well defined and typically contain degenerate A-rich motifs that often lack the U3–A6 Hoogsteen base pair nucleotides. In agreement with this, the U3–A6 binding pocket created by the N-terminal region of WDR33 is not conserved in yeast Pfs2. Conversely, the structure and sequence conservation of yeast Yth1 ZF2 and ZF3 domains, which specifically recognize A bases in higher eukaryotes, suggests that recognition of adenosine dinucleotides by Yth1 is conserved. Structural differences in the RNA recognition modes of the yeast and human machineries could explain the observations that yeast CPF_core_ binds to model RNA substrates with much lower affinity than human CPSF [13^••^,22^••^].

## Mechanism of endonucleolytic cleavage

**Figure 3:**
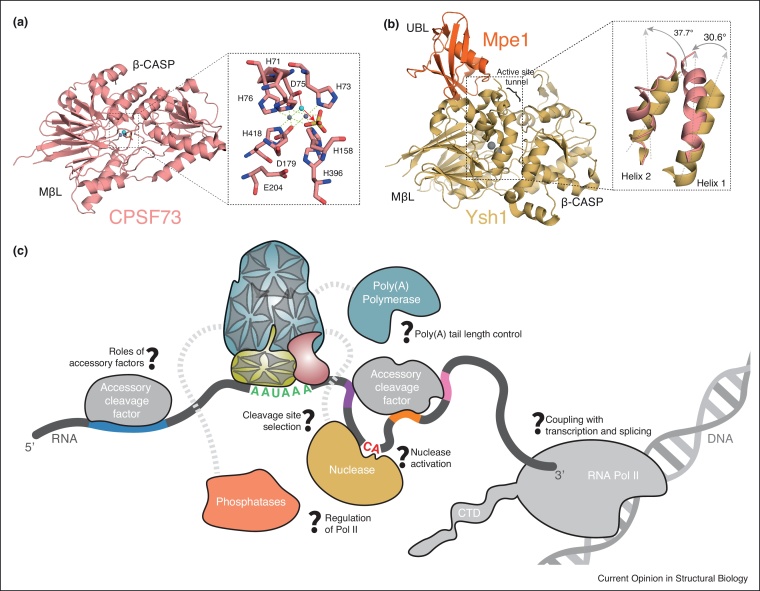
Structures of the Ysh1/CPSF73 endonuclease and model for 3′-end processing. **(a)** X-ray crystal structure of human CPSF73 showing the β-CASP and metallo-β-lactamase (MβL) domains (PDB code 2I7T). Zinc ions are shown as grey spheres and their coordination is indicated with dashed lines. In the active site, a hydroxide ion (shown as a blue sphere) is the attacking nucleophile and a sulfate molecule mimics the phosphate group of an RNA substrate. **(b)** Crystal structure of Ysh1 bound to ubiquitin-like domain (UBL) of Mpe1 (PDB code 6I1D). Inset shows superposition with the CPSF73 structure, revealing movement of two helices that could resemble a pre-activation state where the nuclease is primed for activation. Ysh1 is shown in gold, Mpe1 in orange and CPSF73 in pink. The active site tunnel lies behind the two helices formed by residues 95 to 111 (helix 1) and 122 to 136 (helix 2) of Ysh1 as indicated. **(c)** A model of mRNA 3′-end processing. The polymerase module hub of Cft1/CPSF160, Pfs2/WDR33 and Yth1/CPSF30 is shown with other subunits and factors surrounding it. Accessory cleavage factors are CF IA, CF IB, CstF, CF Im and CF IIm. RNA is colored as in Figure 1. Questions remaining are indicated.

Ysh1/CPSF73, the endonuclease subunit of CPF/CPSF complexes, contains metallo-β-lactamase (MBL) and β-CASP domains. The crystal structure of human CPSF73 revealed an active site at the junction of the two domains containing two coordinated zinc ions [4^•^] (Figure 3a). The geometry of the zinc ions, coordinating a hydroxide ion (the attacking nucleophile) and a sulfate ion (mimicking the scissile phosphate group in the RNA substrate) in the active site, suggests a possible catalytic mechanism (Figure 3a). However, the active site tunnel in this structure is very narrow and cannot accommodate the RNA substrate. In agreement with this, purified human CPSF73 has only weak endonuclease activity *in vitro* [4^•^] and CPSF-dependent pre-mRNA cleavage has not been reconstituted *in vitro* to date. Together, these observations suggest that conformational activation of CPSF73 is required before pre-RNA cleavage. This may be important to prevent spurious nuclease activity and pre-mRNA misprocessing.

Recent biochemical reconstitution of the yeast pre-mRNA cleavage and polyadenylation machinery showed that the Ysh1 endonuclease subunit is only active when assembled into the ∼500 kDa, 8-subunit ‘CPF_core_’ complex [13^••^]. Specific and efficient RNA cleavage also requires the presence of both CF IA and CF IB. Interestingly, the recombinant CPF_core_ complex cleaved RNA substrates *in vitro* within a window of 3 nucleotides suggesting that it has positional accuracy, but does not have strict nucleotide specificity.

To gain insight into mechanisms that may prime Ysh1 for activation within CPF_core_, crystal and cryoEM structures of yeast Ysh1 in complex with the ubiquitin-like (UBL) domain of another CPF subunit Mpe1 (ortholog of human RBBP6) were determined [13^••^] (Figure 3b). These structures revealed an interaction interface burying ∼900 Å^2^ of surface area and involving hydrophobic, polar and electrostatic contacts. Two α-helices within the Mpe1-bound Ysh1 MBL domain are shifted when compared to the CPSF73 structure (Figure 3b). This results in a slight opening of the cavity leading to the active site, but additional conformational changes must occur to accommodate the RNA substrate. The relatively high atomic B-factors of the β-CASP domain suggest that it may be mobile, allowing further opening of the active site cleft upon activation. Nevertheless, the precise mechanism underpinning this process and its dependence on other CPF/CPSF subunits and accessory polyadenylation factors still remains poorly understood.

## Conclusions and future challenges

Recent integrative structural studies have revealed the molecular architecture of the eukaryotic mRNA 3′-end processing machinery. Although these studies provide key insights into the assembly, RNA recognition and enzymatic activities of these factors, a number of questions concerning their molecular functions still remain unanswered (Figure 3c).

A critical aspect of the molecular mechanism of CPF/CPSF concerns the coupling of its RNA binding, endonuclease and poly(A) polymerase activities. A 3-D reconstruction of yeast CPF_core_ obtained by negative-stain electron microscopy indicates that Cft1, Pfs2 and Yth1 of the polymerase module form a structural scaffold onto which Pap1 and the nuclease module are tethered [13^••^]. Conformational dynamics within CPF may serve to accommodate the variable distance between the polyadenylation signals and cleavage sites observed in different pre-mRNAs and might enable remodeling of the complex upon binding CF IA, CF IB and pre-mRNA. It is likely that similar mechanisms exist within the human complex. To understand the molecular basis of nuclease activation, future studies will focus on the structures of CPF/CPSF bound to accessory factors and additional *cis*-acting RNA elements.

Further studies will also be required to understand how the 3′-end processing machinery regulates transcription. It is known that a phosphatase subunit of yeast CPF (Ssu72) dephosphorylates Ser5 of the C-terminal domain (CTD) of Pol II to facilitate the switch from transcription initiation to elongation [31], while Glc7 dephosphorylates Tyr1 to promote transcription termination [32]. Structural studies will define how the phosphatase module interacts with CPF and whether the phosphatases are mechanistically coupled to RNA recognition, cleavage and polyadenylation.

In mammalian cells, up to ∼70% of genes have more than one PAS, giving rise to alternative mRNA isoforms that may encode different protein isoforms, or contain distinct 3′-UTRs conferring different stabilities and translational efficiencies [33]. The CPSF subunits Fip1 and RBBP6, as well as the accessory factors CstF and CF Im, have been implicated in the regulation of PAS selection [34, 35, 36, 37]. Furthermore, interactions between the 3′-end processing factors and the pre-mRNA splicing machinery have also been shown to contribute to alternative polyadenylation [33,38]. The mechanistic basis for alternative polyadenylation remains to be determined.

Finally, mRNA poly(A) tails generally reach a defined length of ∼60 As in yeast [39,40^•^] and ∼250 As in human [41]. Nuclear poly(A) binding proteins (yeast Nab2 and Pab1, and human PABPN1) play roles in regulating the activity and processivity of poly(A) polymerase, thus specifying the poly(A) tail length, but the molecular details of this process are unknown [42]. The recent structural and biochemical studies described here provide a foundation for future investigations that will focus on these aspects of mRNA 3′-end biogenesis and their regulation.

## Conflict of interest statement

Nothing declared.

## References and recommended reading

Papers of particular interest, published within the period of review, have been highlighted as:

• of special interest
•• of outstanding interest
